# Draft Genome Sequence of *Mycobacterium tuberculosis* Clinical Strain G-12-005

**DOI:** 10.1128/genomeA.00385-14

**Published:** 2014-05-08

**Authors:** Jean-Luc Berland, Fabíola Marques de Carvalho, Luiz Gonzaga Paula de Almeida, Nino Bablishvili, Marie Gauthier, Glaucia Paranhos-Baccalà, Ana Tereza Ribeiro de Vasconcelos

**Affiliations:** a Fondation Merieux, Lyon, France; b Laboratório Nacional de Computação Científica, Petrópolis, Brazil; c National Center for Tuberculosis and Lung Disease, Tbilisi, Georgia

## Abstract

Infection caused by drug-resistant *Mycobacterium tuberculosis* is a growing concern, especially in eastern Europe. We report an annotated draft genome sequence of *M. tuberculosis* strain G-12-005 obtained from a patient in Georgia.

## GENOME ANNOUNCEMENT

Although the incidence of tuberculosis is steadily decreasing in Georgia, to 116/100,000 to date (1), the increasing spread of multidrug-resistant tuberculosis (MDR-TB) jeopardizes efforts to reduce the burden of the epidemic. The development of resistance leads to a challenge for patient treatment outcome and endangers the health worker community.

The *Mycobacterium tuberculosis* Beijing lineage is present in Georgia, among other countries, and its frequency is overrepresented within MDR-TB strains at the global level (2). Some authors hypothesized that this lineage favors the mutation rate, which leads to therapy escape (3) or an increased transmission rate (4). Others have raised the influence of the IS*6110* insertion sites on the fitness of the bacilli (5).

We present the draft genome sequence of a strain isolated from a sputum sample from a 42-year-old woman with pulmonary tuberculosis. This strain is genotypically resistant to streptomycin and isoniazid. *M. tuberculosis* G-12-005 sequencing was performed using the Illumina HiSeq system. The assembly was performed by Newbler version 2.8. Of the total sequenced fragments, the reads were randomly selected to allow 100× genome coverage. The scaffolds were obtained by Newbler and the gaps were improved by GapFiller 1.11 (6). The automated annotation was done by BLASTp against the NCBI NR, KEGG, UniProt, and TCDB databases, using 100% query and subject coverage, and a minimum of 95% positive as the cutoff. *M. tuberculosis* H37Rv (accession no. NC_000962.3) was used as a reference genome. Manual annotation was performed using the System for Automated Bacterial Integrated Annotation (SABIA) (7).

The *M. tuberculosis* G-12-005 assembly results in 4.2 Mbp total length, with 65.61% G+C content and an average coding gene length of 953 bp. A total of 104 gaps were closed, and 108 scaffolds with 137 contigs were obtained. The coding regions correspond to 87.46% of the genome. Of these, 2,905 open reading frames showed homology to known proteins, and 1,027 sequences were considered to be hypothetical. According to Bidirectional Best Hits comparison, G-12-005 presents 3,339 clusters in common with H37Rv. According to KEGG, the majority of the coding genes in G-12-005 are associated with amino acid metabolism (16%), carbohydrate metabolism (14.5%), and energy metabolism (10%). The data are available on http://www.labinfo.lncc.br/projects/mtg12005/.

Therefore, the genome of strain G-12-005 is a source for mining data to unravel the genomic factors involved in the development of resistance to antibiotics or increased transmission rate by spotting mutations and insertion sequence positions; it also presents an opportunity to establish a starting point for surveillance and understanding of the transmission chain through the local community.

### Nucleotide sequence accession numbers.

This whole-genome shotgun project has been deposited at DDBJ/EMBL/GenBank under the accession no. JHUF00000000. The version described in this paper is version JHUF01000000.
